# Limited Neutralization of Omicron by Antibodies from the BNT162b2 Vaccination against SARS-CoV-2

**DOI:** 10.21203/rs.3.rs-1518378/v1

**Published:** 2022-04-14

**Authors:** Tobias V. Lanz, R. Camille Brewer, Shaghayegh Jahanbani, William H. Robinson

**Affiliations:** Stanford University; Stanford University; Stanford University; Stanford University

## Abstract

Since early December 2021, the omicron variant has posed additional challenges to the world-wide management of the SARS-CoV-2 pandemic. Immune evasion is a key factor for its increased transmissibility. While serological studies have measured levels of neutralizing antibodies in response to vaccines, our understanding of the humoral immune response to omicron on a single-antibody level is limited. Here, we characterize a set of BNT162b2 vaccine-derived antibodies for neutralization of omicron pseudovirus. We show that approximately 50% of neutralizing anti-RBD antibodies cross-neutralize omicron, albeit with lower potency than the original Wuhan-Hu1 strain. All investigated neutralizing anti-S2 antibodies cross-neutralize omicron, however all of them are less potent than anti-RBD antibodies. While additional booster immunizations of the current vaccine generate increased antibody levels and better protection, we anticipate that the second generation of vaccines will yield more high-affinity antibodies against omicron.

## Introduction

Within less than two months, the B.1.1.529 variant (omicron) of the severe acute respiratory syndrome coronavirus 2 (SARS-CoV-2), replaced B.1.617.2 (delta) as the most dominant strain worldwide. With 37 amino acid substitutions in the Spike protein (S), it marks a major antigenic shift from the original Wuhan-Hu1 and the delta sequence. Immune evasion is a major contributing factor foromicron’s improved transmissibility ^1–3^. To date, all approved vaccines against SARS-CoV-2 are based on the Wuhan-Hu1 sequence of the S protein. Serologic studies have shown that convalescent patient plasma and plasma from individuals vaccinated with BNT162b2 neutralize omicron 44-fold and 12-fold less effectively than delta ^2^. A third immunization with BNT162b2 (booster) increases neutralization efficacy by 10 to 100-fold ^2,4^. Accordingly, the booster prevents 88% of omicron-related hospitalizations, as opposed to only 52% at 25 + weeks post second vaccination ^5,6^. This increased efficacy should be attributed to higher anti-S antibodies with increased affinity to Wuhan-Hu1 – and to omicron only by proxy. While sera and plasma neutralization levels have been established, the anti-omicron response to BNT162b2 is not well understood on a single antibody level.

We recently investigated the acute B cell response to the BNT162b2 vaccine on a single-cell level and discerned the development of anti-receptor binding domain (RBD) and anti-S2 antibodies from naïve and memory B cells, respectively ^7^. The RBD is located on the S1 domain of the S protein and interacts with human angiotensin-converting enzyme 2 (ACE2) to initiate viral cell entry, whereas the S2 domain facilitates viral cell membrane fusion ^8,9^. Most neutralizing antibodies found in COVID-19 patients target RBD ^10^. Neutralizing antibodies against the S2 subunit have been described, but they generally neutralize SARS-CoV-2 less efficiently than anti-RBD mAbs^11,12^. However, we have shown that anti-S2 mAbs develop early in response to vaccination, and are cross-reactive to other betacoronaviruses due to higher structural conservation of the S2 subunit over S1 ^7,13,14^. Anti-S2 antibodies could therefore be crucial for the protection against novel variants. We previously expressed and tested 50 vaccine-derived monoclonal antibodies (mAbs) against RBD/S1 and S2, and identified 15 anti-RBD mAbs that neutralized Wuhan-Hu1 and delta ^7^. Here, we added additional 55 mAbs from the same sequence dataset and investigated their binding to RBD and S2 as well as their neutralization potency to Wuhan-Hu1, delta, and omicron.

## Results

**Figure 1:**
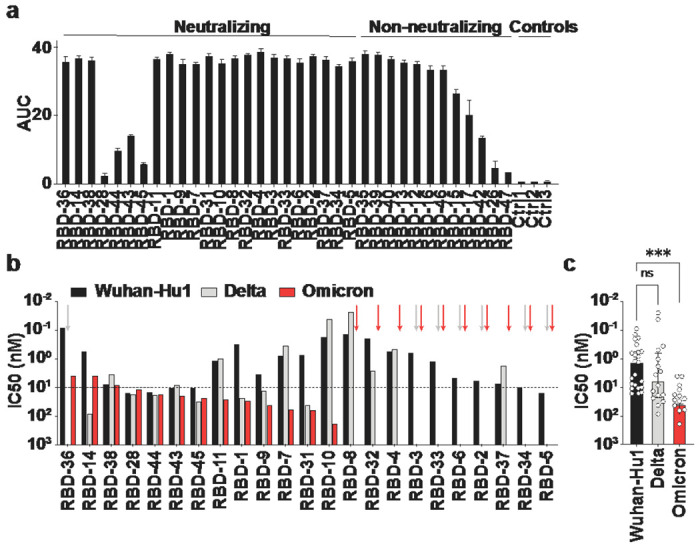
Binding and neutralization of BNT162b2-derived anti-RBD antibodies. **(a)** ELISA measurements of anti-RBD mAbs to Wuhan-Hu1 RBD, mean AUC ± SD. **(b)** Neutralization (IC50) of individual anti-RBD mAbs against Wuhan-Hu1, delta, and omicron pseudovirus. Arrows indicate non-neutralization of the respective antibody against the variant of the same color, dashed line at IC50 = 10nM facilitates comparison with anti-S2 antibodies in Fig. 2b. **(c)** Comparison of IC50 values of anti-RBD mAbs against Wuhan-Hu1, delta, and omicron. Medians ± interquartile ranges are shown; ns, not significant; ***P = 0.0002, unpaired two-tailed Kruskal-Wallis test, Dunn-corrected for multiple comparisons.

Of the 105 included mAbs, a total of 35 bound to RBD (Fig. 1a), of which 23 neutralized Wuhan-Hu1 pseudovirus with variable potency (Fig. 1b,c). 16 of 23 Wuhan-Hu1-neutralizing anti-RBD mAbs neutralized delta and 13 neutralized omicron (Fig. 1b). Neutralization potencies for omicron were decreased 19-fold compared to Wuhan-Hu1 (median half-maximum inhibitory concentration (IC50) Wuhan-Hu1:1.28 nM, omicron: 24.33 nM) (Fig. 1c). The best neutralizing mAb against omicron was 46-fold less potent than the best neutralizing mAb against Wuhan-Hu1 (min. IC50 Wuhan-Hu1: 86 pM, omicron: 4.01 nM). In contrast, overall neutralization potency to delta was only 4.6-fold decreased (median IC50 Wuhan-Hu1: 1.28nM, delta: 5.93 nM) and maximum neutralization potencies were in a similar range for Wuhan-Hu1 and delta (min. IC50 Wuhan-Hu1: 86 pM, delta: 24 pM) (Fig. 1c).

**Figure 2:**
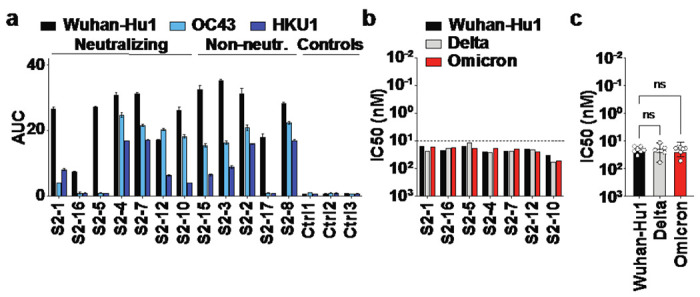
Binding and neutralization of BNT162b2-derived anti-S2 antibodies. **(a)** ELISA measurements of anti-S2 mAbs to Wuhan-Hu1 S2, OC43 S, and HKU1 S, AUC ± SD. **(B)** Neutralization (IC50) of individual anti-S2 mAbs against Wuhan-Hu1, delta, and omicron pseudovirus. Dashed line at IC50 = 10nM facilitates comparison with anti-RBD antibodies in Fig. 1b. **(c)** Comparison of IC50 values of anti-RBD mAbs against Wuhan-Hu1, delta, and omicron. Medians ± interquartile ranges are shown; ns, not significant, unpaired two-tailed Kruskal-Wallis test.

In addition, we identified 12 anti-S2 mAbs, of which 7 neutralized Wuhan-Hu1 pseudovirus (Fig. 2a,b). As previously described, most vaccine-derived anti-S2 antibodies cross-reacted with the betacoronaviruses OC43 and HKU1 (Fig. 1a) ^7^. The anti-S2 mAbs neutralize Wuhan-Hu1 with 16.9-fold lower potency than anti-RBD mAbs (median IC50 anti-RBD: 1.28 nM, anti-S2: 21.60 nM) (Fig. 2b). Interestingly however, all anti-S2 mAbs also neutralize delta and omicron with comparable IC50s, likely due to more conserved epitopes on the S2 subunit (Fig. 2c).

## Discussion

We investigated vaccine-derived antibody neutralization of omicron on a single-antibody level and showed that approximately 50% of anti-RBD antibodies neutralize omicron, albeit with a 19-fold lower potency. Strikingly, the most potent neutralizing antibodies against omicron had a 46-fold decreased potency compared to the best neutralizing antibodies against Wuhan-Hu1. As highly potent neutralizing antibodies are important for immune protection, this difference is substantial, and while additional booster immunizations with the original BNT162b2 vaccine will increase antibody levels, it is unlikely to generate larger amounts of high-affinity anti-omicron-RBD antibodies.

For anti-S2 mAbs, the rate of cross-neutralization against omicron is higher (7 out of 7 mAbs in our study). Anti-S2 antibodies stem from a recall response of memory B cells against prior infections with heterologous betacoronaviruses ^7,13,14^. Their reactivity is broader and less susceptible to immune escape by novel variants. The Evolution of omicron has likely been driven by anti-RBD antibodies rather than anti-S2 antibodies (mutation rate RBD: 7.77/100 amino acids, S2: 1.02/100 amino acids). However, overall neutralization potency is 16.9-fold lower than for anti-RBD antibodies, and anti-S2 antibodies therefore likely contribute limited protection. Vaccine strategies that generate broadly-neutralizing anti-S2 antibodies and therapeutic strategies with high-potency anti-S2 monoclonal antibodies ^14^ have promise to work effectively against novel variants.

In conclusion, our neutralization data on vaccine-derived monoclonal antibodies is in line with serological studies, as we show antibodies with decreased neutralization potency against omicron. Vaccine updates will likely achieve high-potency anti-omicron antibody responses.

## Materials And Methods

### Recombinant expression and purification of monoclonal antibodies (mAbs).

All heavy and light-chain (HC and LC) sequences were obtained by single-cell repertoire sequencing from individuals on days 7, 21, and 28 post initial BNT162b2 vaccination (second immunization on day 21)^7^. Codon-optimized HC and LC variable sequences were cloned into in-house vectors, containing human IgGI and κ or λ constant regions, respectively. HC and LC plasmids were transfected into Expi293F cells using FectoPro (Polyplus transfection). Cell supernatants were collected after 7 days and purified with AmMag Protein A beads (Genscript). mAb concentrations were measured using a nanodrop spectrophotometer (Thermo Fisher Scientific) and human IgG quantitation ELISAs (Bethyl Laboratories).

### ELISA measurements.

MaxiSorp 384-well plates (Thermo Fisher Scientific) were incubated for 24h with 1 μg ml^−1^ recombinant SARS-CoV-2 RBD (Acro, SPD-C52H3), SARS-CoV-2 S2 (Acro, S2N-C52H5), HCoV-OC43 S (Sino Biological, 40607-V08B), or HCoV-HKU1 S (Sino Biological, 40606-V08B), in carbonate-bicarbonate buffer. Plates were washed with PBST (PBS + 0.1 % Tween20) six times between each step. Plates were blocked with PBS + 1 % BSA for 1h at RT, then mAbs were added at concentrations of 10, 1, 0.1, and 0.01 μg ml^−1^ in PBS + 1% BSA and plates were incubated for 24h at 4°C. Plates were incubated for 1h at RT with secondary HRP-conjugated goat anti-human IgG antibodies (Bethyl Laboratories) and developed with TMB substrate (Thermo Fisher Scientific). Development was stopped with 2 N sulfuric acid. Four dilutions of positive-control plasma and secondary antibody only, as well as BSA only served as positive and negative controls. Plates were read on a GloMax Explorer Microplate Reader (Promega). ELISA assays were performed at least twice, in duplicate or triplicate.

### SARS-CoV-2 variants and pseudovirus neutralization Assays.

Variant sequences were obtained from www.gsaid.org. Mutations from Wuhan-Hu1 in S and RBD are described in Supplementary Table 1. Full variant S sequences were codon-optimized and replaced the Wuhan-Hu1 S insert in the SARS-CoV-2 spike plasmid (parent plasmids publicly available from J. Bloom laboratory), which was then amplified in E.Coli and checked for correct insertion and sequence integrity by Sanger-sequencing. Pseudotyped lentiviral particles were generated as previously described ^7,15^. Briefly, LentiX 293T cells in 10-cm tissue culture dishes were transfected 24 h post-seeding using Fugene transfection reagent (Promega) with pHAGE-CMV-Luc2-IRES-ZsGreen-W, lentiviral packaging plasmids (HDM-Hgpm2, HDM-tat1b and pRC-CMV-Rev1b) and wild-type or variant SARS-CoV-2 spike plasmids. 48–60h post transfection, viral supernatants were collected and spun at 500g for 10 min. Lentiviral supernatants were concentrated with LentiX concentrator (Takara) according to the manufacturer’s instructions. Pellets were resuspended in EMEM at approximately 1/100 of the initial amount of media, and stored at −80°C until use. Virus was titrated on HeLa-ACE2 cells, provided by Dennis Burton at the Scripps Research Institute.

Neutralization assays were performed as previously described ^7,15^. Briefly, HeLa-ACE2 cells were seeded at 12,500 cells/well in flat-bottom 96-well plates 20h before viral transduction. mAbs were prepared in EMEM in eight five-fold serial dilutions starting at 50 or 10 μg ml^− 1^, incubated with SARS-CoV-2 pseudotyped virus for 1h at RT, and then added to HeLa-ACE2 cells in the presence of 5 μg ml^− 1^ polybrene (Sigma Millipore). After 48h, luciferase activity was measured using the Britelite plus Reporter Gene Assay System (Perkin Elmer), and read on a GloMax Explorer Microplate Reader (Promega). Neutralization assays were performed 1–3 times in at least triplicates for each dilution. mAbs were considered neutralizing only if a considerable decrease in luminescence was measured at concentrations < 10 μg/ml (Supplementary Figs. 1–2).

### Human subjects.

No human subjects were included in this study. Antibody sequences were derived from our prior study ^7^, which had been approved by the Institutional Review Board of Stanford University (IRB-3780), and complied with the relevant ethical regulations.

### Statistical analysis and software.

Statistical analyses were performed with GraphPad Prism v.9.1.0. Significance levels and statistical tests used are indicated in the figure legends. For analysis of ELISA measurements, the areas under the curves (AUC) of serial dilutions were calculated and AUC ± SEM were presented. For neutralization assays, the half-maximum inhibitory concentration (IC50) for each mAb was calculated using least square regression.

## Supplementary Material

1

## Data Availability

All data pertaining to this manuscript are included in the text, figures, and supplementary information.
