# Supplementary material for: Limited Neutralization of Omicron by Antibodies from the BNT162b2 Vaccination against SARS-CoV-2
Source: Res Sq. 2022 Apr 14:rs.3.rs-1518378. Preprint. [Version 1] doi: 10.21203/rs.3.rs-1518378/v1 (PMC9016652; doi:10.21203/rs.3.rs-1518378/v1)
Supplement: 1 [file 3_SupplementaryInformation_V1.docx]

**Supplementary Information**

Supplementary Table 1

Supplementary Figure 1

Supplementary Figure 2

**Supplementary Table 1.** SARS-CoV2 variants and their mutations in S and RBD.

| **Variant name** | **Greek alphabet name** | **Classification** | **Origin** | **S Mutations** | **RBD mutations** |
| --- | --- | --- | --- | --- | --- |
| B.1.617.2 | delta | Variant of Concern | India | T19R, T95I, G142D, Δ156/157, R158G, L452R, T478K, D614G, P681R, D950N | L452R, T478K |
| B.1.1.529 | omicron | Variant of Concern | South Africa / Botswana | A67V, delH69, delV70, T95I, G142D, delV143, delY144, delY145, delN211, L212I, ins214EPE, G339D, S371L, S373P, S375F, K417N, N440K, G446S, S477N, T478K, E484A, Q493R, G496S, Q498R, N501Y, Y505H, T547K, D614G, H655Y, N679K, P681H, N764K, D796Y, N856K, Q954H, N969K, L981F | G339D, S371L, S373P, S375F, K417N, N440K, G446S, S477N, T478K, E484A, Q493R, G496S, Q498R, N501Y, Y505H |

**Supplementary Figure 1.** Neutralization curves of anti-RBD antibodies

**Supplementary Figure 1.** Anti-RBD neutralization curves, corresponding to data in Figure 1, panels C-E. Titration of mAbs in 5-fold dilutions are shown for pseudoviruses Wuhan-Hu1 (blue), and variants delta (red), and omicron (green). Regression lines have been omitted for measurements in which antibody was not neutralizing.

**Supplementary Figure 2.** Neutralization curves of anti-S2 antibodies

**Supplementary Figure 2.** Anti-S2 neutralization curves, corresponding to data in Figure 1, panel F. Titration of mAbs in 5-fold dilutions are shown for pseudoviruses Wuhan-Hu1 (blue), and variants delta (red), and omicron (green). Regression lines have been omitted for measurements in which antibody was not neutralizing.
